# A Simple Method for Imaging and Quantifying Respiratory Cilia Motility in Mouse Models

**DOI:** 10.3390/mps8050113

**Published:** 2025-10-01

**Authors:** Richard Francis

**Affiliations:** Cilia Research Laboratory, College of Medicine and Dentistry, James Cook University, Townsville, QLD 4814, Australia; richard.francis@jcu.edu.au; Tel.: +61-7-4781-6065

**Keywords:** ciliated airway epithelia, cilia beat frequency, cilia generated flow

## Abstract

A straightforward ex vivo approach has been developed and refined to enable high-resolution imaging and quantitative assessment of motile cilia function in mouse airway epithelial tissue, allowing critical insights into cilia motility and cilia generated flow using different mouse models or following different sample treatments. In this method, freshly excised mouse trachea is cut longitudinally through the trachealis muscle which is then sandwiched between glass coverslips within a thin silicon gasket. By orienting the tissue along its longitudinal axis, the natural curling of the trachealis muscle helps maintain the sample in a configuration optimal for imaging along the full tracheal length. High-speed video microscopy, utilizing differential interference contrast (DIC) optics and a fast digital camera capturing at >200 frames per second is then used to record ciliary motion. This enables detailed measurement of both cilia beat frequency (CBF) and waveform characteristics. The application of 1 µm microspheres to the bathing media during imaging allows for additional analysis of fluid flow generated by ciliary activity. The entire procedure typically takes around 40 min to complete per animal: ~30 min for tissue harvest and sample mounting, then ~10 min for imaging samples and acquiring data.

## 1. Introduction

Evaluating how airway epithelial cilia beat is crucial for understanding how genetic mutations and environmental factors affect mucociliary clearance and lung health [1]. This protocol describes a simple method for high-resolution imaging of airway cilia movement using mouse tracheal tissues and works well for examining ciliary activity in both wildtype or genetically modified mouse models and facilitates rapid evaluation of key parameters such as cilia beat frequency, waveform, and cilia generated flow. This protocol only requires basic skills in tissue necropsy/dissection and video-microscopy and has been successfully utilized by even untrained undergraduate students after a single demonstration session.

Current methods for examining airway cilia dynamics generally fall within two categories: acute ex vivo approaches or extended in vitro culture systems. Ex vivo techniques include visualization of human nasal/airway brush biopsies [2,3,4] or analysis of airway tissue sections [5], while in vitro methods involve growing isolated airway epithelial cells in sheets using air-liquid interface cultures to stimulate their reciliation [6,7]. However, both above techniques have their drawbacks. Airway brush biopsies remove ciliated epithelial cells from their natural tissue environment, which can result in cell injury causing cilia dyskinesia [8], while also only allowing analysis of cilia motion in single isolated cells or clumps, making assessment of cilia generated flow problematic as cilia generated flow requires long lengths of coordinated ciliated epithelial cells to become established (This is why the vast majority of cilia studies only assess cilia beat frequency and not cilia generated flow). Conversely, air-liquid interface culture methods are very reagent intensive and time consuming, requiring a long list of specialized culture consumables/reagents, while often taking 4–6 weeks before cilia are ready for imaging [9].

In contrast, the protocol described here offers a faster and less technically demanding alternative for studying airway cilia motility in mouse models. This ex vivo tracheal preparation maintains epithelial tissue structure and signaling pathways by preserving the native tissue context, making it more physiologically relevant than isolated in vitro cultures. The technique requires no specialized dissection tools or advanced equipment beyond standard video microscopy setups. The benefits of this approach are numerous. It supports a fairly high-throughput analysis of animals/tissues while imaging cilia motion in the sagittal plane, providing the best angle for studying beat patterns, metachronal wave activity, and assessment of cilia generated flow. Finally, this protocol can be modified as needed to investigate how various factors, including drugs, gene changes, environmental exposures, and mucus buildup, affect ciliary motility and coordination.

## 2. Experimental Design

### Materials

All reagents and equipment used for this protocol are listed in Table 1.

## 3. Procedure

### 3.1. Gross Necropsy of Mouse Pluck (~30 min per Mouse)

**Figure 1:**
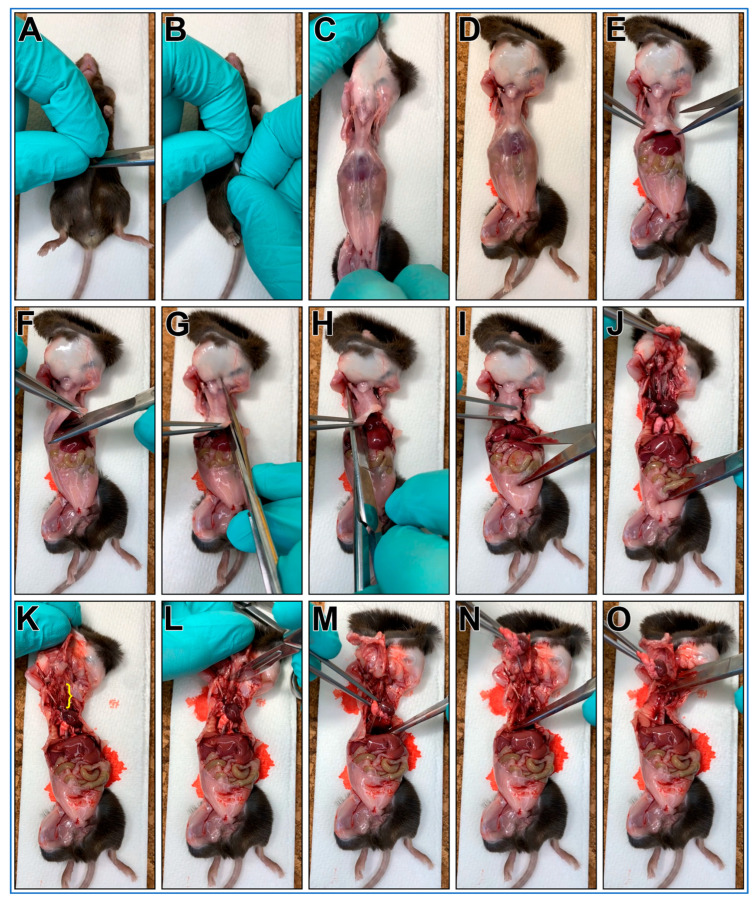
Gross necropsy of a mouse pluck (Heart/Lungs/Trachea). (**A**) Make a small cut through the skin over the abdomen of a freshly euthanized mouse. (**B**) Grab skin on each side of cut. (**C**) Pull skin off mouse abdomen and chest (**D**). (**E**) Using scissors cut through the abdominal wall using a transverse cut just below the diaphragm. (**F**) Grasp xiphoid process with forceps, pull up and make a transverse cut through diaphragm. (**G**) Insert scissor tip into the chest cavity lateral to the sternum (left side) and advance tip up and under the clavicle before cutting. (**H**) Insert scissor tip into the chest cavity lateral to the sternum (right side) and advance tip up and under the clavicle then cut. (**I**) Grasp xiphoid process with forceps, push down on lower body (scissors used in picture). (**J**) pull up with forceps removing the anterior portion of the rib cage. This should reveal the trachea up to the larynx ((**K**), yellow bracket). (**L**) Make a transverse cut through the larynx with a scalpel. (**M**) Grasp heart/lungs with forceps. (**N**) pull pluck up and out of the chest. (**O**) Being careful not to cut into the trachea, cut any remaining small tissue connections with scissors to allow pluck removal.

The steps outlining the gross necropsy of a mouse pluck are shown in Figure 1.

1. A mouse is euthanized via CO_2_ euthanasia and the carcass is placed on ice for transport to the laboratory.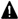 **CRITICAL STEP** Cervical dislocation should not follow CO_2_ euthanasia as it may cause damage to the trachea;
2. The mouse is placed posterior side down onto a cork dissecting board;
3. Dissecting scissors are used to make a small cut through the skin over the abdomen;
4. The skin on each side of the cut are grasped between thumbs/forefingers, then the skin is pulled off the mouse abdomen and chest;
5. **OPTIONAL STEP.** Mouse may be pinned to the dissecting board through each paw. This is recommended for people new to this protocol;
6. Dissecting scissors are used to cut through the abdominal wall using a transverse cut just below the diaphragm;
7. The xiphoid process is secured using blunt forceps to better secure chest for following steps;
8. Dissecting scissors are used to make a transverse cut through diaphragm;
9. One blade of the dissecting scissors is inserted into the chest cavity lateral to the sternum (left side) then advanced up and under the clavicle then cut;
10. One blade of the dissecting scissors is inserted into the chest cavity lateral to the sternum (right side) then advanced up and under the clavicle then cut;
11. The xiphoid process is secured with blunt forceps, then while pushing down on lower body, the forceps are pulled up removing the anterior portion of the rib cage. This should reveal the trachea up to the larynx (Figure 1K, yellow bracket);
12. A transverse cut through the larynx is made with a scalpel;
13. The heart/lungs are secured using blunt forceps, then pulled up removing the pluck up and out of the chest;
14. Being careful not to cut into the trachea, any remaining small tissue connections are cut with scissors to facilitate pluck removal;
15. The mouse pluck is then placed into a 50 mL centrifuge tube with a small amount of DPBS (~3–4 mL).

### 3.2. Fine Dissection of Trachea Sections from the Mouse Pluck

**Figure 2:**
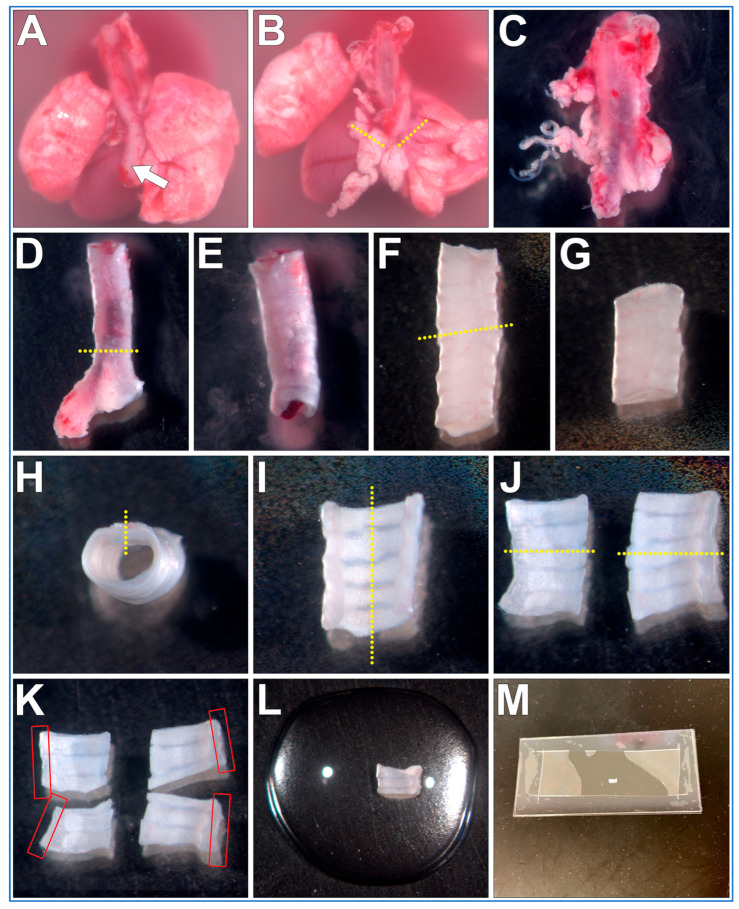
Fine dissection of trachea sections from a mouse pluck for microscope imaging of respiratory cilia. (**A**) Mouse pluck is placed into a 35 mm culture dish filled with DPBS and the oesophagus (arrow) is peeled off the trachea. (**B**) Heart and lungs are removed following transverse cuts through both bronchi (dotted lines). (**C**) Fat/miscellaneous tissue is removed from trachea surface. (**D**) Trachea is cut just above the carina, bronchi tissue discarded. (**E**) Trachea lumen flushed to remove blood/mucus. (**F**) trachea is cut (yellow dotted line), top (superior) length of trachea discarded (**G**). (**H**) A sagittal cut is made through the trachealis muscle (yellow dotted line). (**I**) A second sagittal cut is made through middle of cartilaginous rings (yellow dotted line), resulting in two trachea halves (**J**), which can be imaged, or further sectioned (yellow dotted lines). (**K**) Four trachea sections that can be used for imaging. Cilia are best visualized along the length of the curled trachealis muscle (red boxes). (**L**) Trachea sample placed into a drop of liquid media on top of a glass coverslip. (**M**) Top coverslip/silicon gasket is then lowered onto the bottom glass coverslip/sample forming a shallow walled imaging chamber that can then be imaged using an inverted microscope.

The steps outlining the gross necropsy of a mouse pluck are shown in Figure 2. All the below steps are conducted using a dissection microscope (up to ~10× magnification).

1. The mouse pluck is placed posterior side up into a 35 mm culture dish filled with DPBS;
2. Micro tweezers are used to peel the oesophagus up and off the trachea;
3. Micro scissors are used to make transverse cuts through both bronchi, then the heart and lungs are removed;
4. The isolated trachea is now moved into a new 35 mm culture dish filled with fresh DPBS (old dish inevitability being contaminated with excess blood obscuring details);
5. Using blunt dissection and the micro tweezers/scissors, all fat/miscellaneous tissue is removed from the trachea surface;
6. Micro scissors are used to make a transverse cut just above the carina of the trachea, bronchi are removed and discarded;
7. A P1000 pipette and P1000 tip is used to gently flush the trachea lumen to remove any blood/mucus;
8. Micro scissors are used to make a transverse cut through the trachea ~5–6 cartilaginous rings above the carina. The cranial trachea section is removed and discarded;
9. Micro scissors are used to make a sagittal cut through the trachealis muscle (Figure 2H, yellow dotted line);
10. Micro scissors are used to make a second sagittal cut through the middle of the cartilaginous rings (Figure 2I, yellow dotted line);
11. Tissue can be now mounted and imaged (see below). Cilia are best visualized along the length of the curled trachealis muscle (Figure 2K, red boxes);
12. **OPTIONAL STEP.** Trachea halves can be further sectioned into smaller samples for different experimental treatments (Figure 2K);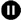 **PAUSE STEP.** Trachea sections can be stored in DPBS at 4 °C for up to 12 h before mounting and imaging [12].

### 3.3. Trachea Sample Mounting (~5 min per Sample)

**Figure 3:**
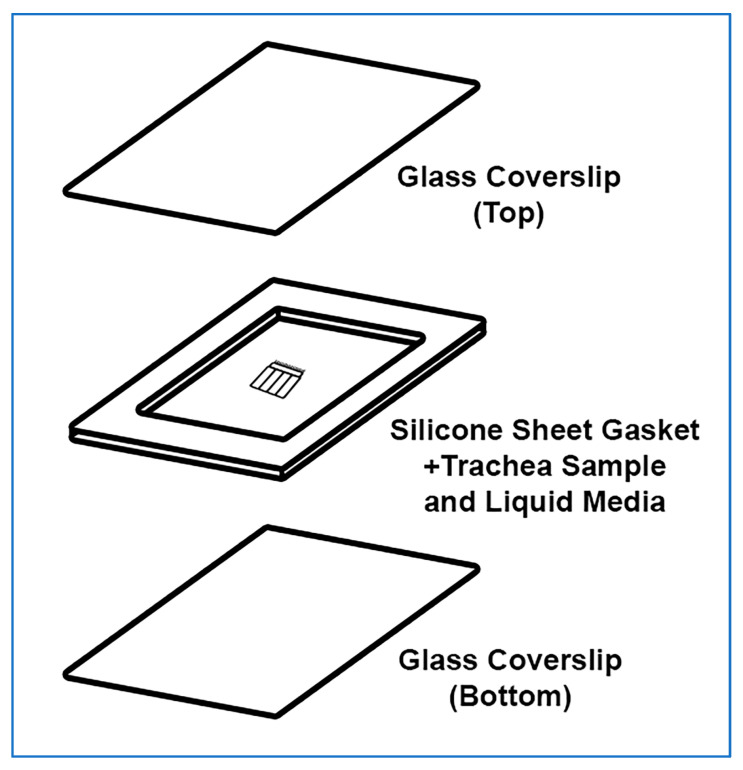
Exploded isometric view of trachea sample mounted for imaging. The top glass coverslip and silicone gasket are assembled first, a drop of liquid media is then placed onto the bottom glass coverslip along with the ciliated trachea sample. The top coverslip is then gently lowered on a 45° angle onto the bottom coverslip and gently pressed in place. No adhesive is required, pressing coverslips and gasket together is sufficient.

The trachea sample mount setup is presented in Figure 3. The power of this technique is that the trachealis muscle curls over when cut, positioning the cilia in a sagittal plane to the microscope objective, with no other tissue above or below, this allows very clear visualization of the cilia waveform allowing very clear measurements of cilia beat frequency and cilia generated flow. Cilia can be imaged in other locations within the tracheal samples, but cilia will not be as clearly visible.

1. A rectangular glass coverslip (24 mm × 50 mm) is covered with ~0.25 mm thick silicone sheet, no adhesive is required, pressing coverslips and gasket together is sufficient;
2. A sharp scalpel is used to remove a small rectangular section of silicone from the middle of the silicone sheet covering the coverslip, excess silicone is also trimmed from the edges of the coverslip as needed;
3. **OPTIONAL STEP.** If silicone sheet is not available, other material of similar thickness may be used to form a gasket (e.g., 1+ layers of electrical tape);
4. A second rectangular glass coverslip (24 mm × 50 mm) is placed onto the stage of a dissection microscope;
5. A drop (~100–200 µL) of DPBS containing 1 μm Carboxylate Microspheres (~1 drop of microspheres per 4 mL of DPBS) is placed onto the middle of the second coverslip;
6. The trachea sample is placed into the middle of the DPBS drop (Figure 2L);
7. The first silicone covered coverslip is gently lowered at a 45° angle onto the bottom coverslip containing the DPBS drop and trachea sample, then gently pressed into place (no adhesive is required, pressing coverslips and gasket together is sufficient); Care is taken so that the silicone layer is between the two coverslips;
8. A Kimwipe or paper towel is used to remove any leaked media from coverslip edges;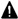 **CRITICAL STEP** Once sample is enclosed within the glass coverslips it should be imaged immediately. The small volume of media and enclosed environment may cause tissue to become hypoxic if left too long, which may impact cilia motility;

The sample is now ready for the microscope.

### 3.4. Cilia Imaging (~5 min per Sample)

1. The trachea sample mounted as above is now placed onto the stage of an inverted bright-field microscope, preferably with Differential Interference Contrast (DIC) filters for optimal cilia visualization and a high magnification/NA water/glycerol or oil immersion objectives (e.g., 63×/1.4);
2. **OPTIONAL STEP.** As the coverslip sample mounts are fragile, a microscope with adjustable slide holder is recommended (e.g., Thorlabs, MZS500P2—MZS500-Compatible Slide/Petri Dish Holder). Samples can then be simply lowered onto the objective without using clips to secure sample in place;
3. **OPTIONAL STEP.** A heated microscope incubation chamber is preferable for these studies, but cells kept at room temperature are still usable for imaging ciliary motility. The major drawback of this would be that cilia in colder temperatures display decreased motility;
4. Standard microscopy techniques are used to move/scan and image cilia within the mounted samples;
5. A microscope camera capable of high-speed imaging (>200 fps) is used to image cilia motility to ensure accuracy of measurements. Camera speeds ~300 fps are recommended, especially if samples are imaged at 37 °C;
6. One second movies are collected at >200 fps for quantification of CBF;
7. Ten second movies are collected at 30 fps for tracking microsphere movement to quantify cilia generated flow.
8. **OPTIONAL STEP.** This protocol may be coupled with fluorescent microscopy and/or other live cell imaging dyes (e.g., fluorescent microspheres for flow assessment). However, care should be taken to minimize bright excitation light exposure which may damage and impair cilia motility (personal observation);

### 3.5. Data Analysis (~10–20 min per Sample)

**Figure 4:**
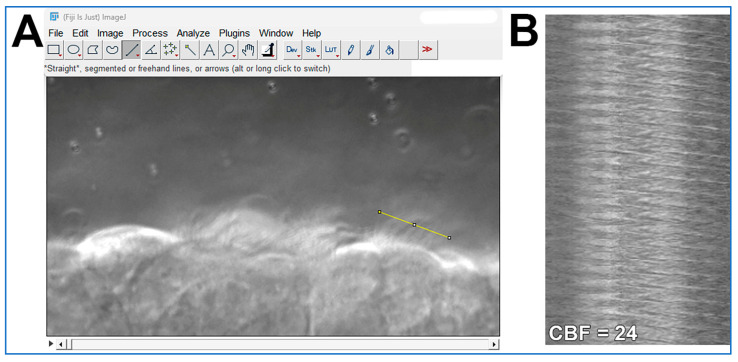
Demonstration of how cilia beat frequency can be calculated from cilia movies using ImageJ software. (**A**) A high-speed movie (.avi format; ~300 fps; 1 s length) of cilia motion is opened in ImageJ. The straight-line tool is used to draw a line through the moving cilia of one epithelial cell (yellow line in **A**). The ImageJ Reslice tool is then used to generate the kymograph image shown in (**B**). (**B**) Kymograph of cilia motion generated from the yellow line in panel (**A**). Each wave in the Kymograph represents one cilia beat. As the movie used in (**A**) is 1 s in length, simple counting of waves yields cilia beat frequency for that epithelial cell = 24 beats/second.

1. Cilia movies are analyzed using ImageJ (FIJI 2.3.0/1.53q);
2. CBF is quantified from the one second movies (>200 fps) as outlined in Figure 4. The ImageJ straight-line tool is used to draw a line through the moving cilia of one epithelial cell (Figure 4A). The ImageJ Reslice tool is then used to generate a kymograph image (Figure 4B). As the movies are one second in length, simple counting of waves yields cilia beat frequency for each ciliated cell analyzed;
3. Cilia generated flow is quantified from the ten second movies (30 fps) by tracking the 1 µm microspheres suspended within the media by using the Manual Tracking plugin for ImageJ (FIJI 2.3.0/1.53q; https://imagej.net/plugins/manual-tracking; accessed on 5 August 2025);
4. Microsphere directionality (ratio representing liner flow) is calculated from the flow data using Microsoft excel by dividing net microsphere displacement by total distance travelled.

## 4. Expected Results

**Figure 5:**
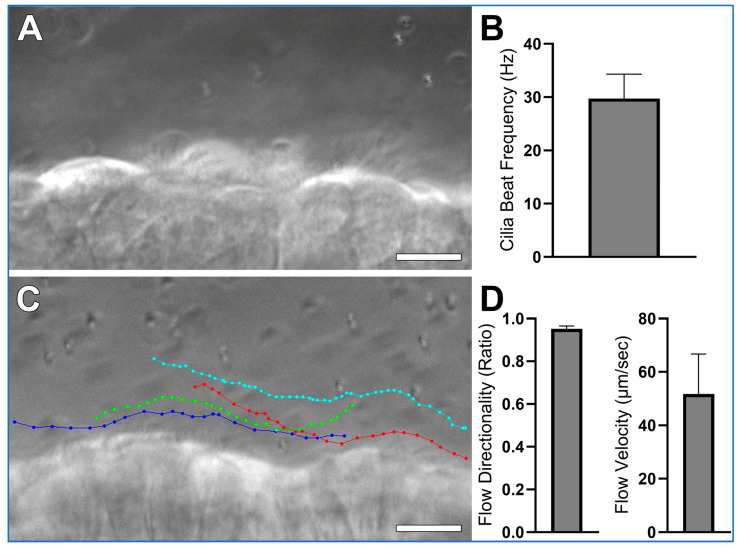
Expected results collected using wild-type mice trachea, prepared according to the protocol outlined in this study. (**A**) A representative still image from a high-speed (~300 fps) differential interference contrast (DIC) recording is shown from which cilia beat frequency is calculated (See Supplemental Video S1, Panel B). (**B**) Mean cilia beat frequency calculated from 4 ciliated cells as outlined in data analysis, cilia beat frequency in this sample is slightly higher than expected mean for wild-type trachea samples which are generally ~20 Hz, this is probably due to the low sample size (n = 1 sample). (**C**) Four coloured traces showing the movement of four 1 µm microspheres across the ciliated epithelial surface (See Supplemental Video S1, Panel A). (**D**) Flow Directionality and Flow velocity were derived from microsphere-tracking data as outlined in data analysis. Results are presented as mean ± SD. Scale bars = 10 μm.

Expected results collected according to this protocol, using wild-type mice trachea, are shown in Figure 5 and Supplemental Video S1. Airway cilia should be readily visible and exhibit synchronized beating (Supplemental Video S1B). This activity generates a detectable directional fluid flow along the axis of ciliary motion (Supplemental Video S1A). Measurements derived from such recordings are expected to be consistent with the data presented in Figure 5. High-speed differential interference contrast (DIC) imaging (Figure 5A) enables accurate quantification of ciliary beat frequency (Figure 5B) and provides sufficient resolution to visually assess ciliary waveform. Meanwhile, 1 µm microsphere tracking (Figure 5C) serves as a reliable method to determine both the velocity and directionality/linearity of cilia-driven fluid flow (Figure 5D). Directionality is calculated by dividing the total displacement traveled by a microsphere (i.e., the shortest distance between the start and end points of the tracing) by the distance the microsphere moves over the whole trace. Thus, microspheres moving in a straight-line display directionality ≈ 1; while microspheres moving randomly display directionality ≈ 0. Hence microsphere velocity reflects cilia generated flow speed, while microsphere directionality reflects the linearity of cilia generated flow.

## 5. Discussion

Measurement of cilia beat frequency (CBF) is relatively straightforward with some simple microscopy/imaging equipment [13] which is why CBF is often the primary/only metric measured in studies examining airway cilia motility in both health and disease [2,14,15]. However, while CBF is certainly informative, it overlooks two key components of ciliary function, namely cilia waveform and the resultant cilia generated flow, which is essential for normal mucociliary transport [16]. These aspects are more challenging to assess so they are frequently overlooked or ignored.

The focus on CBF rather than assessing cilia waveform or cilia generated flow is problematic as perturbations in cilia waveform, which is highly specialized in shape to best facilitate flow generation, can result in defective cilia generated flow while CBF may appear unchanged (i.e., a cilia flapping backwards and forwards at the same rate as cilia with normal waveform will generate less flow). The disconnect between CBF and flow generation can be seen in patients with DNAH11 mutations which display airway cilia with elevated CBF [17]. But despite this increase, they still experience poor mucociliary clearance because cilia waveform is abnormal resulting in impaired mucociliary clearance [17].

This protocol offers numerous improvements over a previously published protocol written by this author [18]. Firstly, this protocol provides an exhaustive step by step walkthrough, which has been previously lacking, including the description of pluck removal, which is not as easy as it seems, especially for new users and students. This technique has been developed over numerous years for undergraduate student teaching and has proven successful even for students that have never done a mouse dissection before. Secondly, several cost saving changes were also made such as using simple glass slides for sample mounting instead of glass bottom culture dishes, and the use of DPBS instead of L15/FBS [18] or other complex media [19] for sample suspension. The discovery that DPBS was just as effective as more expensive media for short term assessment of mouse airway cilia was a recent finding made in my laboratory [12]. The use of a simpler media is beneficial because it reduces the media component experimental variable, allowing easier interpretation of experiments focused on how different chemicals/treatments influence cilia motility. Thirdly, this protocol only requires the use of a simple bright field microscope instead of a fluorescence microscope [18,20] or confocal microscope [21], meaning the protocol will be accessible for a larger number of laboratories. Furthermore, I have also made preliminary observations suggesting that bright excitation light may be damaging to cilia, so the use of brightfield imaging may prove beneficial in maintaining normal cilia motility (although this needs further work to confirm). Finally, with the significant improvement in camera technology over the last decade, this protocol provides several cost-effective camera options for high-speed imaging of cilia motion, including a camera costing <600.00 USD$ at time of publication (1.5 MP USB3.0 Mono Microscope camera E3CMOS01500KMA).

One of the major strengths of this protocol is that it allows airway cilia to be imaged in the sagittal plane, perpendicular to their direction of motion. This profile view gives a much clearer picture of how cilia beat (waveform), not just how fast they beat. It also lets us see the cilia generated flow of fluid across the surface of the epithelium in real time. As a result, we can directly compare beat frequency, waveform, and flow within a single tissue sample, something that’s difficult/impossible to achieve with other techniques.

Another advantage of this protocol is that the freshly dissected tissue retains the ciliated epithelia within its natural tissue environment, preserving possible local cell-signaling pathways. In contrast, airway cultures grown in the lab often struggle to produce uniform ciliation and typically display uncoordinated or random beating patterns [22,23]. The preserved coordination in our ex vivo samples provide a more accurate representation of in vivo conditions and opens the door to studying how cilia coordinate their activity across the tissue.

## 6. Conclusions

In conclusion, this protocol outlines a simple method for collecting, imaging, and quantifying respiratory cilia motility in mouse tissue. By introducing a few straightforward adjustments, this protocol can serve as a versatile approach for investigating how various factors influence airway cilia activity. These include the effects of drugs, temperature variations, genetic modifications, environmental exposures, and physical influences such as mucus burden.

## Figures and Tables

**Table 1:** Tools, reagents, and equipment needed for this protocol.

	Source	Identifier
Tools		
Cork Dissecting Board	Agar Scientific Ltd. (Stansted, United Kingdom)	AGL4121
#10 Scalpel Blades	Roboz Surgical Instrument Co. (Gaithersburg, MD, USA)	RS-9801
No 3. Scalpel Handle	Roboz Surgical Instrument Co. (Gaithersburg, MD, USA)	65-9843
Blunt Dressing Forceps	Roboz Surgical Instrument Co. (Gaithersburg, MD, USA)	RS-8100
Dissecting Scissors; Straight; 5” Length	Roboz Surgical Instrument Co. (Gaithersburg, MD, USA)	RS-6808
Micro Scissors, Straight	Roboz Surgical Instrument Co. (Gaithersburg, MD, USA).	RS-5602
#4 Inox Dumont Tweezers	Roboz Surgical Instrument Co. (Gaithersburg, MD, USA).	RS-4904
Equipment		
Basic Dissection Microscope. Allowing up to ~8–10× magnification. e.g., Leica EZ4 (Leica, Wetzlar, Germany); Zeiss Stemi 305 (Zeiss, Oberkochen, Germany); Olympus SZX7 (Olympus, Tokyo, Japan); Nikon SMZ800N (Nikon, Tokyo, Japan).		
Inverted Bright-Field Microscope, preferably with Differential Interference Contrast (DIC) filters (e.g., Leica DMi8 (Leica, Wetzlar, Germany); Zeiss Axiovert 5 (Zeiss, Oberkochen, Germany); Nikon Eclipse Ts2 (Nikon, Tokyo, Japan)). High magnification/NA (≥40×/>1 NA) water/glycerol or oil immersion objective. Heated incubation chamber (37.5 °C) optional, but lower temperatures will result in slower cilia [10].		
Microscope camera capable of high-speed imaging ^†^. Examples: 1.5 MP USB3.0 Mono Microscope camera E3CMOS01500KMA (ProSciTech Pty Ltd., Thuringowa, Australia); 3.2 MP FLIR Grasshopper3 USB Camera GS3-U3-32S4M-C (Teledyne Vision Solutions, Waterloo, ON, Canada); Phantom Miro C321 (Vision Research Inc., Wayne, NJ, USA).		
Reagents		
Kimwipes (12 × 21 cm)	Thermo Fisher Scientific (Waltham, MA, USA)	25509-KL
Dulbecco′s Phosphate Buffered Saline ^‡^	Sigma-Aldrich (St. Louis, MO, USA)	D8662
35 mm culture dishes	Sigma-Aldrich (St. Louis, MO, USA)	CLS430165
50 mL centrifuge tubes	Sigma-Aldrich (St. Louis, MO, USA)	CLS430829
Rectangular glass coverslips, No. 1, 24 mm × 50 mm	Sigma-Aldrich (St. Louis, MO, USA)	CLS2975245
P1000 Micropipette	Sigma-Aldrich (St. Louis, MO, USA)	FA10006M
1000 μL filtered pipet tips	Sigma-Aldrich (St. Louis, MO, USA)	CLS4809
Immersion oil	Sigma-Aldrich (St. Louis, MO, USA)	56822
1 μm Carboxylate Microspheres	Polysciences (Warrington, PA, USA)	08226-15
0.254 mm thick silicone sheet	AAA Acme Rubber Co. (Tempe, AZ, USA)	010X36-64904

^†^ I have previously shown that camera speed (fps) should be >2.5 times greater than cilia beat to ensure accuracy of measurements [11]. However, to best visualize respiratory cilia waveform, camera speeds >250 fps are recommended, especially if samples are imaged at 37 °C. ^‡^ Other liquid media may be used for suspending/imaging trachea samples. I have previously shown that mouse trachea can be stored/imaged up to 12 h after harvest using most commonly available liquid media (DPBS, HBSS, L15, M199, RPMI, DMEM) with little difference on cilia motility/cilia-generated flow [12].

## Data Availability

The data presented in this study are available on request from the corresponding author.

## Supplementary Materials

The following supporting information can be downloaded at: https://www.mdpi.com/article/10.3390/mps8050113/s1, Video S1: Representative movie showing cilia motion in wildtype mouse tracheal.

## Abbreviations

The following abbreviations are used in this manuscript:

CBF	Cilia Beat Frequency
DIC	Differential Interference Contrast
DMEM	Dulbecco’s Modified Eagle Medium
DPBS	Dulbecco′s Phosphate Buffered Saline
fps	Frames Per Second
HBSS	Hank’s Balanced Salt Solution
L15	Leibovitz’s L-15 Medium
M199	Medium 199
RPMI	Roswell Park Memorial Institute Medium
